# Item generation for a new disease-specific health-related quality of life questionnaire in patients with deep vein thrombosis: an international multicenter study

**DOI:** 10.1016/j.rpth.2026.103443

**Published:** 2026-03-25

**Authors:** Elia Asady, Lars-Petter Jelsness-Jorgensen, Waleed Ghanima, F.A. Klok, Susan R. Kahn, Gudula J.A.M. Boon, Yvonne Ende-Verhaar, Crystal Sequeira, Hilde Skuterud Wik

**Affiliations:** 1 Department of Research, Østfold Hospital Trust, Grålum, Norway; 2 Institute of Clinical Medicine, University of Oslo, Oslo, Norway; 3 Department of Gastroenterology, Østfold Hospital Trust, Grålum, Norway; 4 Department of Health, Welfare and Organisation, Østfold University College, Halden, Norway; 5 Department of Medicine – Thrombosis and Haemostasis, Leiden University Medical Center, Leiden, the Netherlands; 6 Department of Medicine, McGill University, Montreal, Quebec, Canada; 7 Centre of Excellence in Thrombosis and Anticoagulation Care, Jewish General Hospital, Montreal, Quebec, Canada; 8 Department of Internal Medicine, Haaglanden Medisch Centrum, The Hague, the Netherlands; 9 Department of Haematology, Oslo University Hospital, Oslo, Norway

**Keywords:** health surveys, patient-reported outcome measures, quality of life, surveys and questionnaires, venous thrombosis

## Abstract

**Background:**

Deep vein thrombosis (DVT) can lead to persistent physical symptoms and psychological burden that significantly impact patients’ health-related quality of life (HRQoL). Despite the availability of disease-specific HRQoL questionnaires, a recent systematic review found that none fully adhered to current international development guidelines.

**Objectives:**

To develop an item list for a future disease-specific HRQoL questionnaire for patients in the chronic phase after a DVT, with a strong emphasis on capturing patient experiences.

**Methods:**

Following the European Organization for Research and Treatment of Cancer and US Food and Drug Administration guidelines, item generation was based on: (1) review of existing patient-reported outcome measures identified through a prior systematic review; (2) input from clinicians across Norway, Canada, and the Netherlands; and, most importantly, (3) qualitative data from semistructured focus group interviews with patients, which were analyzed thematically using Braun and Clarke’s methodology. The resulting item bank was reviewed to remove duplicates and merge similar concepts by consensus.

**Results:**

Thematic analysis identified 4 major themes: discomfort, pain, limitations in functioning, and psychological impact. Combining interview data with literature-derived items and input from clinicians resulted in a comprehensive item bank of 400 items. After consolidation and review, a final 52-item list was established.

**Conclusion:**

We identified 52 items deemed relevant to patients’ HRQoL in the chronic phase of DVT. By adhering to current guidelines and actively involving patients throughout the development process, we have laid the foundation for a new disease-specific HRQoL questionnaire for this population.

## Essentials

• DVT can impact patients’ HRQoL.
• We aimed to identify relevant issues for a future HRQoL questionnaire.
• Four themes emerged: discomfort, pain, limitations in functioning, and psychological impact.
• We identified 52 issues deemed relevant to patients in the chronic phase of DVT.

## 1 Introduction

Deep vein thrombosis (DVT) and the subsequent development of postthrombotic syndrome impose a lasting burden on patients due to persistent symptoms, treatment, and the psychological and physical impact of the condition [[1], [2], [3]]. Postthrombotic syndrome develops gradually over the years following an acute DVT and occurs in at least 20% to 50% of patients who have had a proximal DVT of the lower limb [4,5]. It involves various chronic manifestations, including pain, heaviness, swelling, itching, skin changes, and venous ulcers [6].

The use of patient-reported outcome measures (PROMs), such as health-related quality of life (HRQoL) questionnaires, gives patients a voice in clinical studies and has become an integral endpoint, or even the primary endpoint, in some studies [[7], [8], [9], [10]]. Several studies have shown that DVT can significantly reduce HRQoL [[11], [12], [13], [14], [15]]. This, together with the increasing understanding of the chronic nature of DVT, has prompted the development of several HRQoL questionnaires for this population [16].

Guidelines for the development of HRQoL questionnaires have been established by the European Organization for Research and Treatment of Cancer (EORTC) and the US Food and Drug Administration [17,18]. These guidelines aim to enhance the overall usefulness and methodological rigor of PROMs, and a modular development guide with 4 phases is recommended: (1) generation of relevant HRQoL issues; (2) conversion of the issues into a set of items; (3) pretesting the item list; and (4) large-scale international field testing [17]. Furthermore, patient participation in the form of qualitative interviews has been highlighted as the most important step to ensure high content validity, ie, the appropriateness of a measure’s content for its target population [17,18]. Indeed, studies indicate that physicians’ assessments of HRQoL can both overestimate and underestimate patients’ experiences [19].

Based on the recommendations and guidelines for the development of PROMs, we recently investigated whether existing disease-specific HRQoL questionnaires used for the assessment of patients after a DVT had been developed in line with these recommendations. Our systematic review concluded that none of the available questionnaires fulfilled all key criteria outlined in existing guidelines, namely patient involvement during development, appropriate methodology, and international collaboration [16].

The aim of this study was to develop a content-valid item list for a future disease-specific HRQoL questionnaire targeting the chronic phase of DVT, with a primary focus on capturing patients’ experiences and perspectives.

## 2 Methods

### 2.1 User involvement

To ensure the involvement of patients in all phases of the questionnaire development, an advisory panel of 3 patients was established to provide appropriate representation while remaining feasible in size for repeated meetings and active participation. The panel was recruited through the outpatient clinic at Østfold Hospital and participated in several face-to-face and online meetings. In addition to a diagnosis of DVT, inclusion criteria were loosely defined to ensure representation of both biological sexes and a range of ages. The advisory panel was involved throughout the study and systematically consulted on several key aspects, including the development of the interview guide, the analytical process, and the definition of the transition time from acute to chronic phase. This transition was set at 3 months after the occurrence of DVT, based on clinical reasoning and patient input.

### 2.2 Overview of the item generation process

**Figure 1:**
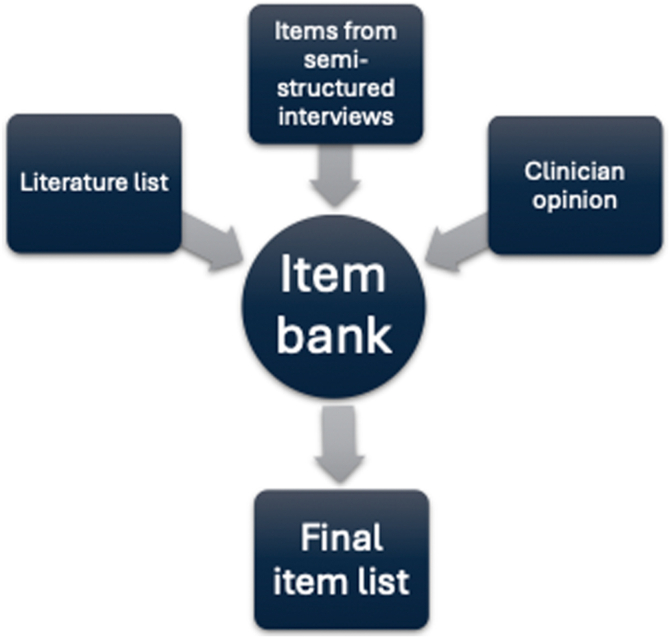
Overview of the item generation process.

To establish and identify a complete item bank for the questionnaire development, 3 steps were used: (1) review of the literature (see section 2.2.1); (2) input from clinicians (see section 2.2.2); and (3) focus group interviews with patients (see section 2.2.3). The items identified through these steps were synthesized into a comprehensive item bank. This bank was then refined and reduced to produce the final item list (Figure 1).

#### 2.2.1 Review of the literature

In step 1, the results of our previously published systematic review [16], which identified 6 PROMs, were used to extract a list of items based on existing PROMs. This list of items from the literature was developed by merging all items from existing PROMs into a comprehensive set. Each item was then condensed to its primary concept; for example, the question “During the past 4 weeks, have you had any pain in your ankles or legs, and how severe has this pain been?” was compressed to “Pain in the ankles or legs.” This was done to standardize and simplify the various question formats. Exact duplicates were removed, while items exhibiting slight variations in wording or context were retained for further evaluation.

#### 2.2.2 Input from clinicians

In step 2, the list of items from the literature served as a compilation of issues previously published as relevant to patients with DVT, which was presented to clinicians for evaluation. Clinicians were asked to review each item and indicate whether they considered it relevant to patients with DVT and, if not, why? In addition, they were invited to suggest any additional issues they believed were missing from the list.

#### 2.2.3 Focus group interviews with patients

In step 3, DVT patients who accepted the invitation to participate took part in roundtable, semistructured group interviews conducted in Norway, Canada, and the Netherlands.

#### 2.2.4 Creating the item bank and final item list

A comprehensive item bank was first created by combining lists of items from the literature, clinician opinions, and focus group interviews. The initial list included all items from each source without filtering and therefore contained substantial overlap and duplicate items. In the next step, this item bank was reviewed by the research team to reduce redundancy and overlap. Exact duplicates were removed, and similar items were merged by consensus among E.A., H.S.W., L.-P.J.-J., and W.G. Items that did not emerge during the interviews and were considered irrelevant by patients were reviewed by the team and considered for removal by consensus. Items considered potentially distinct by any team member were retained for further evaluation, forming the basis of the final item list.

### 2.3 Recruitment and data collection

Physicians and nurses were recruited through professional networks in Norway, the Netherlands, and Canada, and invited to review the list of items from the literature electronically. Members of the main research team in each country contacted professional colleagues with expertise in thrombosis and invited them to complete the questionnaire online. No specific eligibility criteria were applied beyond regular clinical involvement in the treatment of patients with thrombosis.

Patients were recruited from hospitals in Norway, Canada, and the Netherlands. Patients were eligible for inclusion if they were diagnosed with DVT of the lower limb and/or pelvic veins by radiological tests >3 months prior and were aged ≥18 years. Exclusion criteria included severe comorbidities such as dementia, recent stroke, or severe neurological diseases. Additionally, patients with comorbidities other than DVT causing symptoms in the lower limbs, eg, significant skin disease, sequelae from previous trauma or fractures, congestive heart failure with leg edema, severe obesity, severe peripheral artery disease, or arthritis, were excluded. Patients unable to understand or complete the questionnaires were also excluded from participation.

Patients from Østfold Hospital were recruited through its venous thrombosis registry, an ongoing, single-center registry of consecutive patients with venous thromboembolism who were diagnosed, treated, and/or followed up at Østfold Hospital, Norway. The registry was established in 2005 and covers a population of approximately 317,000 inhabitants [20]. Patients from Oslo University Hospital were recruited from the outpatient clinic. Patients recruited through the venous thrombosis registry and outpatient clinics were randomly selected from different time points in the registry to ensure representation of patients at varying stages since diagnosis. The patients’ clinical records were screened by clinicians to determine eligibility. In the Netherlands, patients were recruited by approaching an online venous thrombosis patient group, where an advertisement was published inviting patients to participate in the interviews. In addition, eligible patients visiting the outpatient clinic of the Haaglanden Medisch Centrum were asked to participate. In Canada, patients were recruited from the outpatient clinic. Clinical records were screened to ensure eligibility.

Consent forms, demographic data, and the patient-reported Villalta score [21] were collected prior to interview start. After completion of the interviews, patients received the list of items from literature. After reviewing the list, patients were asked whether any items reminded them of issues not discussed during the interview.

While patients from Norway and the Netherlands received a paper version of the list of items from literature, patients in Canada reviewed the list of items from literature electronically. Each patient was able to review the item list privately and without interruption.

### 2.4 The interview guide and process

The interview guide was developed following the general guidelines outlined by EORTC and Richard Krueger’s work to standardize interviews and assist moderators throughout the preparation and interview processes [17,22]. Moderators were encouraged to remain neutral, facilitate discussions, and guide the interview effectively (Supplementary Methods). Moderators did not have any clinical or personal relation to the patients.

Moderators informed patients about the project and the voice recording, as well as the possibility to engage moderators individually at the end of the interview. They were also instructed to avoid any direct or suggestive questions, while probing questions were encouraged.

To ensure consistency across interviews, 6 main questions were designed. The first question, “How has DVT affected your life?” explored the general impact of DVT on patients’ lives, encouraging open discussion. This was followed by broad questions addressing the effects of DVT on physical health, mental health, social life, work, and home situations.

Each focus group in Norway and the Netherlands was planned to include 4 to 9 patients. The interviews in Canada were conducted online and with fewer patients for each interview due to the COVID-19 pandemic. Native-speaking moderators from the local study team performed the interviews.

### 2.5 Statistical analysis

The data obtained from the interviews were analyzed using thematic analysis according to Braun and Clarke’s 6-phase approach [23]. First, the audio recordings from the interviews were transcribed verbatim by the interviewing moderator. The transcripts were then carefully reviewed multiple times by the research team, enabling thorough understanding and initial reflections.

In the second phase, initial codes were systematically generated from the transcripts, representing meaningful features of the data. Examples included verbatim codes such as “worried about bleeding.” Coding focused primarily on semantic content without deeper interpretation. To ensure coding reliability and consistency, 2 researchers (E.A. and H.S.W.) independently coded 1 interview transcript. Subsequently, coding results were compared, confirming agreement and similarity in coding patterns.

In the third phase, the research team examined the initial codes, sorting and collating them into broader patterns to identify potential themes. This involved grouping similar codes together, exploring relationships, and developing preliminary thematic maps.

During the fourth phase, the identified themes were reviewed in detail, focusing first on coherence within themes and subsequently on the validity and accuracy of these themes across the entire dataset. This process was iterative, involving continuous refinement through revisiting the original transcripts, reassessing groupings, and adjusting as necessary to ensure thematic coherence and integrity.

The fifth phase involved further analysis, clearly defining each theme to capture the essence and main characteristics of the underlying data. Theme labels were thoughtfully created to concisely and accurately represent their content.

Finally, the sixth phase encompassed the selection of representative and compelling extracts from the transcripts to illustrate and support the identified themes. These extracts were embedded in a comprehensive analytic narrative, effectively conveying the thematic patterns and addressing the research questions.

### 2.6 Ethical considerations

This study was approved by the ethics committees of Norway, the Netherlands, and Canada, and written informed consent was obtained from all patients.

## 3 Results

### 3.1 Review of literature

A total of 83 items were identified and included in the list of items from literature.

### 3.2 Input from clinicians

Forty-three clinicians participated in the evaluation of items identified from the literature, including 11 from Norway, 16 from the Netherlands, and 16 from Canada. Of these, 38 were physicians with experience in DVT treatment ranging from 2 to 41 years (mean, 16.9 years), and 5 were specialized nurses with experience ranging from 2 to 18 years (mean, 8.4 years). Clinicians reviewed each item for relevance to the DVT patient population. Less than 50% of respondents rated the following items as relevant: stumbling, bruising, restless legs, and feeling on edge. The remaining items were considered relevant by >50% of respondents. No additional items were suggested by the clinicians.

### 3.3 Interviews

A total of 48 patients participated in the semistructured group interviews (*n* = 23 in Norway; *n* = 18 in the Netherlands; *n* = 7 in Canada). Of these, 46 provided demographic data (Table 1). Sixty-five percent of patients were female, despite equal recruitment efforts for both sexes.

**Table 1:** Baseline characteristics of patients in semistructured group interviews for the development of a health-related quality of life questionnaire for deep vein thrombosis.

Characteristic	*N* = 46, *n* (%)
Age (y), mean (SD; min-max)	54.9 (15.1; 21-81)
Sex	
Male	16 (34.8)
Female	30 (65.2)
Marital status	
Single	5 (10.9)
Divorced or widow/widower	8 (17.4)
Married, living together, or in a relationship	33 (71.7)
Living alone	
No	38 (82.6)
Yes	8 (17.4)
Highest education	
Elementary school	2 (4.3)
High school	8 (17.4)
Secondary vocational education	14 (30.4)
Higher (professional) education	22 (47.8)
Job	
Full-time	22 (50.0)
Part-time	7 (15.9)
Student	2 (4.5)
Retired	8 (18.2)
Unemployed	3 (6.8)
Other	2 (4.5)
Smoking	
Never	27 (58.7)
Quit	17 (37.0)
Yes	2 (4.4)
Current use of anticoagulation	
No	18 (39.1)
DOAC	25 (54.3)
VKA	3 (6.5)
Patient-reported Villalta score	
No PTS	9 (23.7)
Mild PTS	15 (39.5)
Moderate PTS	10 (26.3)
Severe PTS	4 (10.5)

No PTS: patient-reported Villalta score of 0 to 4; mild PTS: Villalta score of 5 to 9; moderate PTS: Villalta score of 10 to 14; severe PTS: Villalta score of ≥15 or the presence of a venous ulcer.

DOAC, direct oral anticoagulant; PTS, postthrombotic syndrome; VKA, vitamin K antagonist.

**Figure 2:**
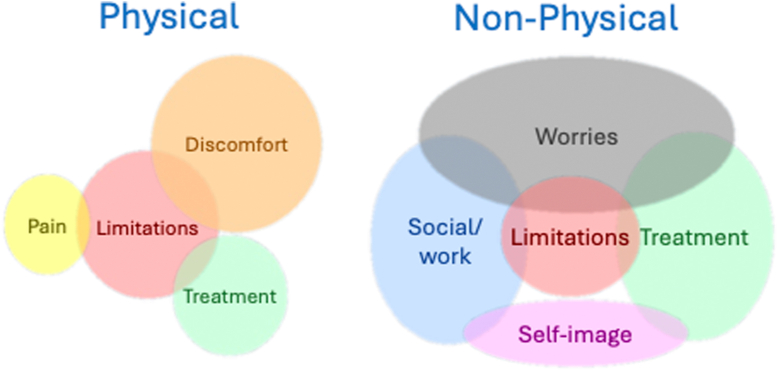
Example of a preliminary thematic map. The overlap represents the interpretation of items that overlap within themes.

The thematic analysis resulted in 4 main themes: (1) discomfort, (2) leg pain, (3) limitations in functioning, and (4) psychological impact. Figure 2 illustrates an example of a preliminary thematic map developed during the analysis.

#### 3.3.1 Discomfort

Discomfort emerged as a prominent theme among physical items. Patients clearly distinguished discomfort from pain, emphasizing continuous, unpleasant sensations without sharp pain. For example, one patient clearly noted, “It’s not pain! It’s just continuous discomfort, but I can’t call it pain. For me this is very different.” Patients often specified, “I have discomfort, like stiffness and heavy legs, not pain,” when the moderators probed this distinction. Cramps were reported to occur at night, during physical activity, or when not using direct oral anticoagulants. For instance, one patient described, “Sometimes I get cramps in my leg at night,” while another remarked, “I have been bothered by cramps for many years, but when I started with Xarelto, I haven’t had a single cramp.” Swelling was a persistent problem, even in those using elastic stockings, as one patient noted: “My leg is swollen daily actually, despite the stockings.” Another explained, “If I stop using stockings for 2 days, my leg starts swelling, and the skin gets tight.” Despite the discomfort of wearing stockings during hot weather and their inconvenience, many patients continued to use them for the symptom relief they provided. Stiffness was also a recurring issue, with 1 patient stating, “My legs are stiff if I’m a long time in front of the computer,” while others experienced stiffness following physical activity.

#### 3.3.2 Pain

Pain emerged as a distinct theme after iterative refinement. Patients described sensations ranging from pain during activity to severe burning or radiating pain in the leg. For instance, one patient described, “When I sit too long and don’t move, the pain is there — no swelling, just like a burning sensation.” Another noted activity-related pain: “Occasionally, when I walk too much, I will have a little pain in the leg when I come back.” The pain significantly restricted physical activities and hobbies for many patients. One patient stated, “I used to ride horses, but this is impossible now because of the pain.”

#### 3.3.3 Limitations in functioning

The theme “Limitations in functioning” underwent the most significant evolution during analysis, initially overlapping substantially with other themes. Through repeated analysis cycles, multiple related codes were grouped, positioning “Limitations in functioning” centrally within the thematic structure. This is well represented in Figure 1, where limitation is at the center of both physical and nonphysical themes. One patient shared, “I have a job where I can get into dangerous situations. I think about it, and I have to be careful since I use blood thinners.” Another noted, “I stopped working as a flight attendant due to the thrombosis.” An active mountain biker explained, “I have lost so much power in my leg, which is very noticeable every time I get on my bike.” The impact on family planning was also significant, with one patient stating, “Because the risk of a new DVT during pregnancy was very high. Therefore, you start thinking about it, and what it’s worth. I decided we won’t have children.”

#### 3.3.4 Psychological impact

Psychological impact first emerged as an overarching theme encompassing several others. Patients described many aspects as a cause of psychological impact. As limitations in functioning absorbed large parts of other themes, psychological impact emerged as a distinct entity. One patient shared, “I think for me the one thing that’s been more affected is my mental state, because let’s say I get a slight cramp in my leg and I think maybe it’s another blood clot, and I kind of get anxiety and stress with that.” Another reflected, “They even looked for cancer. That was very frightening and caused a lot of stress and had a major impact on me.” One patient expressed persistent worry, “I am very afraid the DVT may come back to bite me. I am very anxious, and it is always on my mind.” Changes in body image and clothing due to leg appearance or the use of elastic stockings were also noted.

#### 3.3.5 Patients’ reflections on the interview and the list of items from literature

No further information was forthcoming after patients completed the list of items from literature following the interviews. Most items listed were spontaneously mentioned during the interviews, with a few exceptions, such as hassle with monitoring of anticoagulation or dietary restrictions. A few other items were dismissed by patients as irrelevant to thrombosis, such as stumbling, falling, or cutting yourself more than usual.

### 3.4 Creating the item bank and final item list

The semistructured interviews yielded 317 items representing all issues mentioned by patients prior to any filtering, merging, or removal of duplicates. These were combined with the 83 items from the literature. As no additional issues were suggested by clinicians, this resulted in a comprehensive item bank comprised 400 items, as illustrated in Figure 1. Several items from the literature, such as “stumbling, falling, or cutting yourself,” and those related to dietary restrictions, monitoring, and venous ulcers, were considered irrelevant by patients; following discussion with the research team, 26 items were removed. Some items, including “pain” or “cramps,” were not further specified by patients and were therefore considered too vague, leading to the removal of 39 items by consensus. Exact duplicate items were removed, resulting in the exclusion of a further 121 items. Items with minor linguistic variation, such as “heavy sensation in the legs” and “feeling heavy in the legs,” were merged by consensus, resulting in the removal of 96 additional items. The remaining 118 items were reviewed in detail by the research team and subsequently condensed into a final item list comprising 52 items, presented in Table 2.

**Table 2:** Final item list generated following patient interviews, expert opinion, and a literature search.

1. Pain in the leg when resting
2. Pain in the leg during activity
3. Pain in the leg after sitting for a long time
4. Pain radiating down the leg (pain moving down the leg)
5. Stiffness and soreness in the thigh
6. Chest pain during activity
7. Sensation of heavy legs
8. Leg cramps during the day or after activity
9. Leg cramps at night
10. Fatigue (tiredness)
11. Have to be more careful to avoid injury or exhaustion (more than usual)
12. Altered body image (the way you see, feel, or think about your body) or self-esteem
13. Had to alter the choice of clothing
14. Feel helpless
15. Emotional distress since the blood clot
16. Felt a lack of understanding from other people regarding blood clot symptoms
17. Had a restricted ability to work since the blood clot
18. Had restrictions on what you can do at work
19. Social activities have been restricted
20. Family activities have been restricted
21. Felt limited because of the blood clot
22. Traveling has been restricted because of a blood clot
23. Difficulty breathing since the blood clot
24. Discomfort in the leg during activity
25. Limitations in daily activities
26. Limitations in physical activity
27. Worried about own health
28. Worried about the family’s health
29. Dizziness and concentration problems
30. Leg swelling
31. Itching in the leg
32. Discomfort in the leg after sitting for a long time
33. Discomfort in the leg
34. Financial difficulties because of the blood clot
35. Reduced strength in the leg
36. Been more dependent on family
37. Been unable to take care of family
38. Difficulty walking uphill or up the stairs
39. Restless feeling in the leg
40. Felt tired in the leg
41. Had anxiety
42. Had a leg ulcer
43. Physical activity has been limited
44. Worried about the risk of bleeding
45. Side effects due to blood clot treatment
46. Discomfort due to compression stockings
47. Struggled with blood clot treatment
48. Been aware of “the leg being there” and bothered by it
49. Bothered by discoloration in the leg due to thrombosis
50. Worried about the risk of new blood clots
51. Worried about the cause of the blood clot
52. Other health issues that have been affected by a blood clot in the leg or its treatment

## 4 Discussion

This study identified 52 items deemed relevant to patients in the chronic phase after a DVT, generated through a patient-centered, multistep development process.

Discomfort and pain emerged as dominant, closely related themes in the thematic analysis. This is consistent with previous research, in which both discomfort and pain are frequently reported and described by patients [[24], [25], [26], [27]]. Patients in our study often discussed these experiences jointly but also made a clear distinction between them, highlighting that discomfort and pain, while related, are perceived as qualitatively different. This finding is similar to that of Engeseth et al. [26], in which patients also specifically distinguished pain from discomfort during the interviews.

The theme “Limitations in functioning” captured a wide range of impacts on patients’ daily lives and overlapped substantially with other themes. Limitations in functioning are commonly reported in other studies and are often listed under themes such as psychological restrictions, pain, and discomfort [2,26,28]. Owing to the significant number and variety of patient-reported restrictions, it was considered a distinct theme in line with previous findings [29].

Psychological impact varied substantially among our patients. While some reported significant distress, others were minimally affected. Several qualitative studies have described similar results with a wide range of psychological and emotional impacts in the DVT population [26,28,30,31]. Of note, Højen et al. [32] showed that adolescents and young adults with venous thromboembolism had a persistently higher risk of psychotropic drug purchase compared with matched population controls, further highlighting the psychological impact on this population.

A strength of our study is its robust methodology, which follows the EORTC guidelines and involves patient participation throughout the process to ensure high content validity. The international scope of our study enhances the generalizability of the findings by capturing diverse patient experiences across different healthcare systems and cultures. However, it could be argued that Norway, the Netherlands, and Canada have similar cultures and advanced healthcare systems. This limitation was identified during the study’s planning, and to partially mitigate it, issues identified through our systematic review, drawing on studies from a wide range of cultural and healthcare settings, were incorporated into the item pool.

The current study was planned and initiated prior to the COVID-19 pandemic, but data collection was influenced by the pandemic situation. In Canada, for instance, interviews were conducted online as compared with Norway and the Netherlands. This may, of course, have affected the depth of engagement compared with in-person interviews. Moreover, the online advertisement and recruitment process in the Netherlands may have resulted in the inclusion of patients with a higher burden of chronic complications after DVT. On the other hand, this may also have resulted in a broader range of reported issues, which aligns with the study’s objective of capturing a comprehensive item bank.

Another potential limitation is the subjectivity involved in combining codes during analysis. Although the process was conducted through consensus among authors to minimize bias, the interpretation and merging of codes inherently relied on the researchers’ judgment, which may have influenced the final categorization.

During the item reduction process, some items seemed to be associated with conditions other than DVT. For instance, chest pain during activity might indicate angina, and difficulty breathing could be related to pulmonary embolism. However, as the most important aspect of this study was to place patients at the center of the evaluation and ensure high content validity, these items were retained to allow patients to determine their relevance in the next phase of the study.

Further research is needed to evaluate and refine the relevance of the 52 identified items, ensuring their suitability and comprehensiveness for inclusion in the final questionnaire. Subsequent validation studies should involve larger-scale patient input to determine item relevance, specificity, and relative importance, ultimately facilitating the creation of an effective, disease-specific quality of life instrument for the DVT patient population.

In conclusion, 52 items deemed relevant to patients’ HRQoL in the chronic phase of DVT were identified. By adhering to current guidelines and actively involving patients throughout the development process, we have laid the foundation for a new disease-specific HRQoL questionnaire for this population.
