# Supplementary material for: Item generation for a new disease-specific health-related quality of life questionnaire in patients with deep vein thrombosis: an international multicenter study
Source: Res Pract Thromb Haemost. 2026 Mar 25;10(3):103443. doi: 10.1016/j.rpth.2026.103443 (PMC13134022; doi:10.1016/j.rpth.2026.103443)
Supplement: Appendix [file mmc1.docx]

Supplementary Methods

Interview guide for HRQoL- phase 2

General overview

The interviews will be conducted with ,4-9 participants, 1 moderator and an assistant moderator. The moderator will hold a short presentation, then start with the general topics (deducted from the WHO definition of QoL) and ask the participant to provide their experiences and reflections. After the last general topic, the participants will be able to share any other issues. They will then each receive a copy of the “item list” and be asked to reflect on each of these aspects in terms of relevance and if they have additional aspects to share after reading the item list.

Participants

- Carefully recruited, as to avoid patients with severe systemic illness, psychological illness or dementia.
- Patients with DVT no earlier than 3 months prior to the interview.
- >18yo
- 4 to 8 participants per group.

Environment

- Comfortable
- Circle seating
- Tape recording
- Interview lasting between 60 – 120 min.
- refreshments should be offered
- Pen and paper for each participant
- Prepare name cards (only first name) to be given to each participant and placed on the table.

Moderator

- Exercise mild unobtrusive control
- Adequate knowledge of topic
- Alert and free from distractions
- Has the discipline of listening
- Familiar with questioning route
- Use subtle group control (Expert patients, Dominant talkers, Shy participants, Ramblers)
- For dominant participants, acknowledge their opinions and turn attention to others such as “thank you, what do other people think”
- Use purposeful small talk and create a warm and friendly environment.
- Make a short and simple introduction.
- Use pauses and probes. 5 second pauses. Probes such as “would you explain further?” ”would you give an example?”
- The moderators should remain neutral and listen carefully to everyone with patience. No hint of disapproval, judgment or disagreement should be sensed by the participants. Avoid responses such as “that’s good” or “excellent”.
- Conclude/summarise long/unclear input by participants, then ask if anything was missed.
- To allow participation for everyone it’s important to invite every participant to share, in an orderly manner. At the same time, it’s important to keep in mind that it’s not a strict rule and in case other participants would like to share, or if they seem like they have something to say they should be allowed to share as soon as possible and out of turn. The interviewer then should go back to the order of participation.
- Flexible and spontaneous. If the conversation leads to unexpected but relevant outcomes, allow it to play out.
- The moderators should allow participants to speak freely and intervene as little as possible. Interventions should be limited to cases where intervention is necessary to allow time for every participant and to prevent derailing.

Assistant moderator

- Takes careful notes
- Monitor recording equipment.
- Should be in the lookout for participants that may have something to say and allow for their participation.
- Logistical support during the interview.
- Make note if someone was interrupted, and try to return to that point.

Interview steps:

1. A short introduction starting with the moderator and assistant moderator then the participants.
2. Presentation: this should include a short presentation on thrombosis, the project and the WHO definition of quality of life, and a clear guide on how the interview will proceed.
3. Starting the interview with the first general topic, each participant will have the opportunity to share if the topic is relevant to them, and if so, how?
4. After the last general topic, the participants can share if there are any other issues that come to mind that have not been covered.
5. At the end each participant receives a copy of the “item list” with a short intro into what that list is. They will have a few minutes to read and think about the issues. Then they will be asked if the issues are relevant.
6. All things considered. Ask the participants to reflect on the session. Is there anything else?
7. The moderator returns to the start of the presentation. Reviews the purpose of the study and then summarizes each question quickly and after each question redirects to the participants and asks “can we add anything else to this topic?

General rules and guidelines.

- No right or wrong answers. You may or may not have experienced some or most symptoms.
- The interview is being recorded. Please speak clearly one at a time.
- Please turn off your phones.
- My role as the moderator will be to guide the discussion.
- Please talk freely and to each other.
- We will be available at the end of the interview if you have anything else to share.

The General Topics

WHO defines Quality of Life as an individual's perception of their position in life in the context of the culture and value systems in which they live and in relation to their goals, expectations, standards and concerns. It is a broad ranging concept affected in a complex way by the person's physical health, psychological state, personal beliefs, social relationships and their relationship to salient features of their environment.

DVT may affect people in different ways. You may or may not be affected by what we

mention here. Nevertheless, it’s just as important to let us know if you are not affected.

- How has DVT affected your life?
- How did DVT affect your physical health? (eg, symptoms, activity, training, ulcers)
- How did DVT affect your mental health? (worrying, mood, self-image)
- How did DVT affect your social life?
- How did DVT affect your work/home situation?
- Anything else?

Lastly

The moderators should separate and allow for individual conversations with participants, this is to allow for participants to share in private what they don’t feel comfortable sharing in a group.

Systematic analysis

1. During the interview:
   - 1. The moderators should make sure to clarify comments from participants.
     2. Summarize the participants input and ask if the summary reflects the input
2. Immediately after the interview:
   - 1. Make sure the recording is good.
     2. Make a diagram of the seating. In front of each name note the “items” mentioned. (this could be done during the interview as well)
     3. Moderators summarize the meeting and compare notes.
3. Within the first 24hrs.
   - 1. Listen to the tape and transcribe.
     2. Make notes if something is strongly emphasised or if someone was interrupted.
     3. Share the transcript between moderators and ask for feedback/correction.
4. At the end of the interviews, within days.
   - 1. Categorize the “items” mentioned during the interviews.
     2. Make sure you have reached the point of saturation during the last interview, where no new information was discovered.
     3. Evaluate the need for more interviews?
5. Finally, prepare the report.
   - 1. Summarize an item list and If possible note the number of times each item was mentioned (by different individuals).
     2. Note the demography of participants and summarize.
     3. Share the report with the research group for verification.
     4. Finalize the report.
